# *EphB2* SNPs and Sporadic Prostate Cancer Risk in African American Men

**DOI:** 10.1371/journal.pone.0019494

**Published:** 2011-05-16

**Authors:** Christiane M. Robbins, Stanley Hooker, Rick A. Kittles, John D. Carpten

**Affiliations:** 1 Division of Integrated Cancer Genomics, Translational Genomics Research Institute, Phoenix, Arizona, United States of America; 2 Section of Genetic Medicine, Department of Medicine, Pritzker School of Medicine, The University of Chicago, Chicago, Illinois, United States of America; 3 Section of Hematology/Oncology, Department of Medicine and Institute of Human Genetics, University of Illinois at Chicago, Chicago, Illinois, United States of America; Duke University, United States of America

## Abstract

The *EphB2* gene has been implicated as a tumor suppressor gene somatically altered in both prostate cancer (PC) and colorectal cancer. We have previously shown an association between an *EphB2* germline nonsense variant and risk of familial prostate cancer among African American Men (AAM). Here we set out to test the hypothesis that common variation within the *EphB2* locus is associated with increased risk of sporadic PC in AAM. We genotyped a set of 341 single nucleotide polymorphisms (SNPs) encompassing the *EphB2* locus, including known and novel coding and noncoding variants, in 490 AA sporadic PC cases and 567 matched controls. Single marker-based logistical regression analyses revealed seven *EphB2* SNPs showing statistically significant association with prostate cancer risk in our population. The most significant association was achieved for a novel synonymous coding SNP, TGen-624, (Odds Ratio (OR)  = 0.22; 95% Confidence Interval (CI) 0.08–0.66, p = 1×10^−5^). Two other SNPs also show significant associations toward a protective effect rs10465543 and rs12090415 (p = 1×10^−4^), OR = 0.49 and 0.7, respectively. Two additional SNPs revealed trends towards an increase in risk of prostate cancer, rs4612601 and rs4263970 (p = 0.001), OR = 1.35 and 1.31, respectively. Furthermore, haplotype analysis revealed low levels of linkage disequilibrium within the region, with two blocks being associated with prostate cancer risk among our population. These data suggest that genetic variation at the *EphB2* locus may increase risk of sporadic PC among AAM.

## Introduction

Prostate cancer (PC) remains the most common male specific malignancy diagnosed in the U.S. In 2010 alone about 217,730 new cases of prostate cancer accounted for 28% of diagnoses for the ten leading cancer types. Furthermore, an estimated 32,050 deaths are attributed to this disease annually [1]. Many risk factors such as diet, lifestyle, hormones, age, and race have been implicated as contributing to the risk of prostate cancer; however, family history is the single most significant and reproducible risk factor known, where men with two or three first degree relatives with prostate cancer had a five and 11-fold increased risk of developing prostate cancer, respectively [2], [3].

It is widely known that the incidence rate of prostate cancer varies widely by ancestry with African American Men (AAM) having among the highest prostate cancer rates in the world. The 2002–2006 incidence rate of prostate cancer in the U.S. per 100,000 men was 231.9 in AAM versus 146.3 in European American Men (EAM) [1]. This disparity is seen even more drastically when looking at the death rates amongst these two groups with AAM having a greater than two-fold higher (56.3 per 100,000) prostate cancer death rate compared to EAM (23.6 per 100,000) during this same time-frame [1]. Although it is generally accepted that socio-economic factors are major drivers of the disparity in prostate cancer incidence and mortality, biological factors may also play a role. Recent evidence supports a genetic component contributing at least in part to this racial disparity and influencing the disease progression [4]. Compelling evidence shows strong genetic association between prostate cancer risk and genetic markers at 8q24, where several of the risk alleles have minor allele frequencies that are higher within populations of recent West African decent, suggesting a role in the increased incidence of prostate cancer in AAM [5], [6], [7], [8], [9], [10].

Furthermore, several genes implicated as hereditary prostate cancer tumor suppressor genes such as *HPC/ELAC2 and RNASEL* have been linked to increased risk of prostate cancer in African Americans [11], [12], [13], [14]. To this end, our group has looked at the relationship between somatically altered tumor suppressor genes in prostate cancer and risk of familial prostate cancer among African American men. The EPHB2 tyrosine kinase was first reported as a prostate cancer tumor suppressor gene, harboring somatic mutations in prostate tumors [15]. Furthermore, somatic alterations at the *EphB2* locus have been reported in colorectal cancer as well [16], [17], further supporting a role for *EphB2* as an important cancer gene. Additionally, a germline *EphB2* nonsense variant (3055A>T; K1019X) was positively associated with risk of familial prostate cancer in African American men from high-risk families [18].

Although a previous association study of the *EphB2* gene has focused on coding region variants in familial prostate cancer cases, we have set out to determine if genetic variation at the *EphB2* locus is associated with risk of sporadic prostate cancer in African Americans. Here we report the genotyping of 341 single nucleotide polymorphisms (SNPs) encompassing the entire *EphB2* locus in a population of AAM including 490 sporadic cases and 567 matched controls to search for association between *EphB2* genotypes or haplotypes and risk of prostate cancer in our study population.

## Results

To test for association between genetic variation at the *EphB2* locus and risk of sporadic prostate cancer among AAM, we performed a detailed case/control candidate gene association study. To be comprehensive, rather than using a tagged SNP approached we chose to genotype all known SNPs encompassing the *EphB2* gene within our population. We chose 355 SNPs that had been selected for genotyping by the International HapMap Project in order to develop our own haplotype map of the *EphB2* gene in our specific population. These SNPs were supplemented by a set of 14 *EphB2* coding SNPs previously discovered and reported by our group [18]. After assay development we ended up with a total of 341 SNPs for analysis, which were genotyped in our sample of 1,057 AAM including 490 sporadic cases and 567 matched controls. Details of the sample population can be found in Table 1. To minimize errors potentially caused by population substructuring, all individuals within our sample have been genotyped with an independent set of 100 admixture informative markers (AIMs). An initial analysis was carried out to identify SNPs that were out of Hardy-Weinberg equilibrium (HWE). A total of 26 SNPs departed from HWE (Table S1). Of the remaining 315 SNPs, 19 of them were mono-allelic in our population.

**Table 1:** Clinical characteristics of prostate cancer cases and controls

Characteristics	Controls	Cases	P
Number of participants [n (%)]	567	490	-
Age in years [mean (SD)]	65.9 (10.5)	65.9 (9.3)	0.40
PSA in ng/ml [mean (SD)]	1.9 (1.1)	85.4 (392.4)	<0.001
Gleason score [n (%)]			
<8	-	210 (42.9)	-
≥8	-	280 (57.1)	-
Global west African ancestry [mean (SD)]	0.79 (0.1)	0.82 (0.1)	0.001

**Figure 1:**
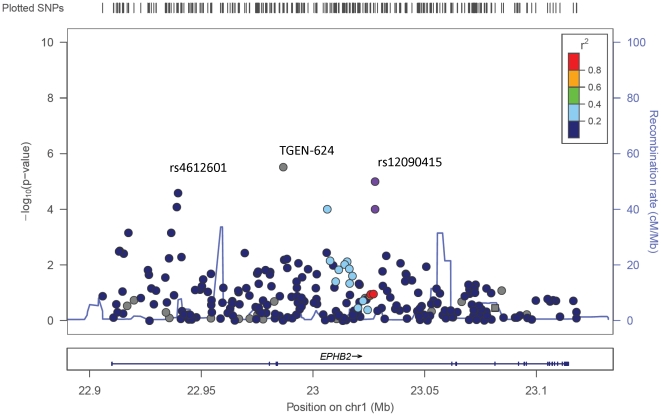
Plot of empirical -log10(p-values) from tests of association between SNPs across *EphB2* gene region and prostate cancer risk in African Americans. Plot includes a display of linkage disequilibrium (r^2^) based on HapMap Yoruba (YOR) data and positions of SNPs along chromosome 1. SNPs shown in gray were not in HapMap. Recombination rates are depicted by the blue line within the plot. Association analysis P-values were adjusted for age and global west African ancestry and visualized using LocusZoom (http://csg.sph.umich.edu/locuszoom/).

Detailed results of the association analysis for all informative SNPs are presented in Table S2. These results are further illustrated in Figure 1. Following corrections for genetic ancestry and multiple testing, we found statistically significant associations for seven SNPs within the *EphB2* gene, which remained significant after multiple testing (Table 2). The most significant association was achieved for a novel synonymous coding SNP, TGen-624, (Odds Ratio (OR)  = 0.22; 95% Confidence Interval (CI) 0.08–0.66, p = 1×10^−5^), which seems to provide a protective effect. Two other SNPs show significant associations toward a protective effect including rs10465543 and rs12090415 (p = 1×10^−4^), OR = 0.49 and 0.7, respectively. Two additional SNPs showed trends towards an increase in risk of prostate cancer, including rs4612601 and rs4263970 (p = 0.001), with OR = 1.35 and 1.31, respectively.

**Table 2:** Allele frequencies and association with prostate cancer for significant *EphB2* SNPs

dbSNP ID	Position (bp)	Gene Region	Allele	Cases	Controls	OR	95%CI	*P*-value
rs4263970	22936517	Intron 1	A	0.51	0.42	1.31	1.16–1.90	0.001
rs1318720	22939099	Intron 1	G	0.26	0.35	0.70	0.54–0.92	0.001
rs4612601	22939631	Intron 1	G	0.56	0.47	1.35	1.18–1.92	0.001
TGEN-624	22983969	-	A	0.01	0.05	0.22	0.08–0.66	1×10^−5^
rs10465531	23006379	Intron 3	A	0.15	0.22	0.71	0.51–0.94	0.004
rs10465543	23006805	Intron 3	A	0.15	0.23	0.49	0.36–0.74	0.0001
rs12090415	23027818	Intron 3	A	0.30	0.40	0.70	0.59–0.93	0.0001

OR [95% CI] and *P*-value adjusted for age and West African ancestry.

Chromosome positions according to Build 36, hg18.

We also tested the possibility of whether these seven SNPs were each independent risk factors or whether the associations were dependent on each other. We performed a stepwise regression using each SNP individually as causal while controlling for the others. None of the other SNPs contributed to the association when either rsTGEN-624 or rs12090415 were considered predictors of risk.

Very little extended linkage disequlibrium (LD) was observed across the *EphB2* gene (Figure 1). Instead we observed 38 small haplotype blocks (largest spanning about 20 kb). This is consistent with the findings that populations of recent West African descent have less LD and smaller block structures (32). Haplotype association analyses were consistent with our single SNP analyses. Only three of the SNPs tested were within defined haplotype blocks (rs1318720, rs4612601, and rs10465531). Haplotype block 6, was tagged by SNPs rs138720 and rs4612601, and haplotype block 18, tagged by SNPs rs12074138 and rs10465531, were significantly associated with prostate cancer (Table 3).

**Table 3:** *EphB2* haplotype frequencies in three major blocks

Block	Haplotype Frequency	Frequency(Case, Control)	*X* ^2^	P-Value
Block 6 rs1318720, rs4612601				
AG	0.496	0.555, 0.464	11.59	0.0007
GA	0.316	0.260, 0.345	11.55	0.0007
AA	0.185	0.182, 0.187	0.05	0.8229
Block 18 rs12074138, rs10465531				
AG	0.573	0.590, 0.565	0.89	0.3456
GG	0.232	0.257, 0.223	2.17	0.1401
AA	0.194	0.151, 0.211	8.09	0.0044
Block 19 rs12133964, rs1440576, rs4655117				
GGG	0.394	0.403, 0.388	0.33	0.5632
GAG	0.365	0.394, 0.355	2.20	0.1377
AGA	0.188	0.147, 0.204	7.47	0.0063
GGA	0.045	0.050, 0.043	0.41	0.5211

## Discussion

The *EphB2* gene encodes the EPHB2 receptor tyrosine kinase and was previously identified by our group as a tumor suppressor gene in prostate cancer [15]. An independent study looking at 72 probands from African American hereditary prostate cancer families identified 10 sequence variants in the *EphB2* gene [18]. This included a common nonsense mutation K1019X that showed an association with PC in AA men that had a family history of PC even after adjusting for admixture. However this association was not seen in AA sporadic cases with PC [18].

Here, we undertook a study to determine if genetic variation at the *EphB2* locus is associated with risk of sporadic prostate cancer among African American men. Not with standing, our comprehensive analysis of the *EphB2* locus further supports the importance of the *EphB2* gene as a putative tumor suppressor gene involved in the disease etiology of prostate cancer and that both common and rare *EphB2* variants likely play a significant role in increased disease risk of sporadic prostate cancer in AAM. The previously identified novel synonymous coding SNP TGen-624 did not show any association of increased risk in AA men with family history of PC, however here we show that in AA men with sporadic disease there is a statistically significant association with risk. This further supports the importance of the *EphB2* gene in not only AAM with familial PC but also in AAM with sporadic PC, although further validation in other datasets is needed to fully support our association.

While we are encouraged by the consistency between our current and previous results, we do note that our study is limited. First, power calculations were used to define the size of our study population, and our modest sample size is sufficient to detect moderate to large effects (OR>1.5). However, we would be limited to detect loci with smaller effects (1.2–1.4). Furthermore, although we genotyped a relatively dense set of known SNPs (∼1 SNP per 700 bp), it is possible that additional novel ‘private’ SNPs with important effects may have been missed within the *EphB2* locus, supporting possible association study designs based upon whole genome or candidate region sequencing to capture the full amount of genetic variation within a given study population.

We captured much of the variation along the *EphB2* gene and observed many small haplotype blocks across the gene, which suggests that the discovery of genetic associations in populations of recent west African descent should rely on high resolution haplotype maps based on African populations. Therefore, genome-wide association studies using array technologies that are based on European haplotype-tagging SNPs or even those with one million evenly spaced SNPs (one SNP per 3 kilobases) may not capture the amount of genetic information needed to detect associations in genes such as *EphB2* in populations of recent west African descent such as ours. We feel this study sets the stage for a new era in genome-wide association studies in populations of recent west African descent, where associations might only be detected through extremely high-resolution genetic maps.

## Materials and Methods

### Ethics Statement

All samples were initially collected for research purposes under a Howard University IRB-approved protocol. Written informed consent was obtained by all subjects for this Howard University study. These samples were then analyzed at the Translational Genomics Research Institute under a protocol approved by Western Institutional Review Board (WIRB). As the samples had already been collected for the Howard University study, a waiver of consent was granted by WIRB for use of the samples in this protocol without additional consent from study participants.

### African American sporadic prostate cancer cases and controls

Unrelated men (N = 1,057) self-described as African American were recruited between the years 2001 and 2005 from the Division of Urology at Howard University Hospital (HUH) in Washington, DC. Incident prostate cancer cases (N = 490) were identified by urologists within the division or study coordinator and confirmed by review of medical records. Control subjects (N = 567) unrelated to the cases and matched for age (±5 years) were also ascertained from the PC screening population of the Division of Urology at HUH. Individuals who were ever diagnosed with benign prostatic hyperplasia (BPH) and/or had an elevated prostate specific antigen test (>2.5 ng/ml), or have had an abnormal digital rectal examination (DRE) were not included as controls. The response rate among the African American cases was 90% and the response rate for the African American controls was 85%. The demographic characteristics of participants in the screening program were similar to the patient population seen in the Division of Urology clinics (Table 1). Recruitment of prostate cancer cases and controls occurred concurrently and were unselected with respect to family history. All participants were between 40 and 85 years of age. Clinical characteristics including Gleason grade, PSA, age at diagnosis and family history were obtained for all cases from medical records. Disease aggressiveness was defined as “Low” (Gleason grade <8) or “High” (Gleason grade ≥8).

### Single Nucleotide Polymorphism (SNP) genotyping

In this study we selected all SNPs (n = 355) encompassing the entire *EphB2* genomic locus between the Build 36, hg18 positions of chr1:22,904,000 - 23,119,000 that were genotyped by the International HapMap (version 2) [19]. An additional 14 SNPs were included that were previously reported by our group [18]. This set of 369 SNPs was submitted for Illumina GoldenGate oligo assay pool (OPA) design, where duplicate SNPs and those that will potentially adversely affect the performance of the assay were removed. A set of 28 SNPs failed assay design, leaving us with 341 SNPs for genotyping in our study (Table S1). Genomic DNA was obtained from isolated lymphocytes using cell lysis, proteinase K-treatment, protein precipitation and DNA precipitation. DNA stocks were diluted to 10 ng/ul and genotyping was performed using the Illumina GoldenGate genotyping assay as per manufacturer's recommendations (Illumina Inc., San Diego, CA). Genotype calls were made using BeadStudio 3 software (Illumina Inc., San Diego, CA). SNPs were excluded from the analysis if the GenTrain Score was <0.4 and the call rate was <0.95. We used a Hardy-Weinberg equilibrium cut-off of p<0.0001 because of increased admixture disequilibrium in the AA population and to take into account multiple testing. Twenty-six (26) SNPs significantly departed from HWE in our African-American samples.

### Estimating genetic ancestry

The genomes of admixed populations such as African Americans are comprised of different genetic segments arising from different “parental” populations (e.g. West Africans and Europeans). Genetic association studies in admixed populations can be confounded by population stratification in which false-positive disease associations arise due to ancestry differences in cases and controls. In order to control for such confounding, West African ancestry was estimated in cases and controls using genetic variants called ancestry informative markers (AIMs). AIMs are markers selected based on their frequency differences between populations from different geographic regions. In the present study, “global” individual ancestry was determined for each individual using 100 AIMs selected from regions across the entire genome to estimate European and West African ancestry [20]. Using these AIMs, global individual ancestry (% West African and % European) was calculated from the genotype data using the Bayesian Markov Chain Monte Carlo (MCMC) method implemented in the program STRUCTURE 2.1 [21]. STRUCTURE 2.1 was run under the admixture model using prior population information and independent allele frequencies. The MCMC model was run using K = 2 populations (58 Europeans and 62 West Africans) and a burn-in length of 30,000 iterations followed by 70,000 replications. These ancestry estimates were used as covariates in the regression models.

### Structured Association Analyses

We tested 315 *EphB2* SNPs for association with PC in our population of African Americans. We calculated odds ratios and 95% confidence intervals by performing conditional logistic regression assuming an additive effect (on the log scale) of allele dosage. We controlled for individual admixture by including West African ancestry estimates as a covariate in the logistic regression model. In addition, genetic effects were adjusted for age (at time of diagnosis for case subjects and at time of ascertainment for controls). Associations by grade (Gleason score ≤7 versus ≥8) were also examined by logistic regression in case-only analyses.

Empirical p-values, which corrected for multiple tests were generated by 100,000 permutations of the trait values in the sample using the Max (T) procedure. All analyses were done using the programs SNPGWA Version 4.0 (http://www.phs.wfubmc.edu/public/bios/gene/downloads.cfm) and PLINK [22]. Haplotype association analyses were performed using Haploview [23]. Clinical characteristics were compared between cases and controls by race. Two-sided t-tests were used to compare continuous variables including age and ancestry estimates. Pearson chi-square tests of independence were used to compare categorical variables.

## Supporting Information

Table S1 Hardy-Weinberg Equilibrium P-values for 341 *EphB2* SNPs.
(XLS)

Table S2 Results of Single Variant Prostate Cancer Association Analysis for 315 *EphB2* SNPs.
(XLS)
